# Profiling Living Bacteria Informs Preparation of Fecal Microbiota Transplantations

**DOI:** 10.1371/journal.pone.0170922

**Published:** 2017-01-26

**Authors:** Nathaniel D. Chu, Mark B. Smith, Allison R. Perrotta, Zain Kassam, Eric J. Alm

**Affiliations:** 1 Microbiology Graduate Program, Massachusetts Institute of Technology, Cambridge, Massachusetts, United States of America; 2 Center for Microbiome Informatics and Therapeutics, Massachusetts Institute of Technology, Cambridge, Massachusetts, United States of America; 3 OpenBiome, Medford, Massachusetts, United States of America; 4 Department of Civil and Environmental Engineering, Massachusetts Institute of Technology, Cambridge, Massachusetts, United States of America; 5 Department of Biological Engineering, Massachusetts Institute of Technology, Cambridge, Massachusetts, United States of America; Wageningen Universiteit, NETHERLANDS

## Abstract

Fecal microbiota transplantation is a compelling treatment for recurrent *Clostridium difficile* infections, with potential applications against other diseases associated with changes in gut microbiota. But variability in fecal bacterial communities—believed to be the therapeutic agent—can complicate or undermine treatment efficacy. To understand the effects of transplant preparation methods on living fecal microbial communities, we applied a DNA-sequencing method (PMA-seq) that uses propidium monoazide (PMA) to differentiate between living and dead fecal microbes, and we created an analysis pipeline to identify individual bacteria that change in abundance between samples. We found that oxygen exposure degraded fecal bacterial communities, whereas freeze-thaw cycles and lag time between donor defecation and transplant preparation had much smaller effects. Notably, the abundance of *Faecalibacterium prausnitzii—*an anti-inflammatory commensal bacterium whose absence is linked to inflammatory bowel disease—decreased with oxygen exposure. Our results indicate that some current practices for preparing microbiota transplant material adversely affect living fecal microbial content and highlight PMA-seq as a valuable tool to inform best practices and evaluate the suitability of clinical fecal material.

## Introduction

Fecal microbiota transplantation—the transfer of fecal microbes from a donor to a patient—has emerged as an extremely effective therapy for recurrent infections of *Clostridium difficile* (~90% cure rate), a common hospital infection that kills nearly 30,000 patients each year [1,2]. Fecal transplants also hold promise for treating other gastrointestinal diseases, like inflammatory bowel disease and irritable bowel syndrome, and even systemic diseases linked to the gut microbiota, like obesity [3]. To date, however, fecal transplants have been proven effective only for recurrent *C*. *difficile* infections, and clinical trials using fecal microbiota transplants to treat inflammatory bowel disease [4–9], irritable bowel syndrome [10,11], and insulin resistance [12] have produced mixed results.

One of the greatest challenges in fecal microbiota transplantation is variability of therapeutic material, which stems from both biological variation and variation introduced by sample handling. Unlike pharmaceuticals, human stool—its microbial and chemical content—varies widely between people and between samples from the same person [13,14]. Many diseases, however, are associated with specific microbes and chemicals [13], suggesting that the composition of a fecal transplant could influence clinical efficacy and side effects. Living microbes are believed to be the therapeutic agent in fecal microbiota transplants [15], since these microbes colonize the recipient patient, potentially leading to lasting changes in the patient's gut bacterial community [16]. As a result, transplant preparation, transportation, and administration—which may kill certain bacteria—could affect clinical efficacy, and best practices are actively debated [3, 17–20]. For example, although current standard practices involve aerobic preparation, exposure to oxygen is known to alter the viability of fecal bacteria, given that most species are obligate anaerobes [17].

In the case of recurrent *C*. *difficile* infection, such presumed aerobic degradation apparently has little impact on clinical efficacy [2]. For other indications, however, where the therapeutic component is poorly understood, variance in living bacteria could significantly affect clinical efficacy. For example, fecal microbiota transplant trials in ulcerative colitis showed fourfold differences in efficacy among different donors, suggesting that specific bacterial communities play a crucial role [9].

We sought to characterize the impacts of typical transplant preparation methods on living fecal microbial communities. We found that freeze-thaw cycles and lag time did not greatly alter the community composition of living bacteria, but oxygen exposure during sample mixing did have a significant effect on the viability of different bacteria. In addition, our results validate PMA-seq as a useful tool for comparing fecal microbiota samples.

## Main

To understand how transplant handling might alter fecal microbial communities—which may affect therapeutic efficacy—we investigated three potential sources of degradation: oxygen exposure during homogenization, freeze-thaw cycles during transplant storage and transport, and lag time between defecation and transplant preparation. For each experiment, we prepared two separate stool samples from the same donor and divided each sample into subsamples for analysis under different transplant preparation methods, thus controlling for variance across fecal samples. After transplant preparation, we then further divided each subsample into three technical replicates. We used qPCR to estimate total 16S rRNA abundance. We then evaluated the replicates' resulting microbial composition using standard 16S rRNA sequencing [21,22] and PMA-seq, which selectively sequences DNA from bacteria with intact cell membranes—a proxy for living cells [23–25].

From our sequencing data, we generated two tables of operational taxonomic units (OTU), one with 1,362 OTUs clustered at 97% similarity (S1 Data) and another with 77 high-confidence OTUs—ones present in all sequencing samples—clustered at 100% similarity (S2 Data).

### Oxygen exposure during fecal homogenization alters the composition of living fecal bacteria

To test the effects on fecal bacteria of oxygen exposure during stool sample homogenization, we prepared subsamples from two stool samples from a single donor using five different procedures, each with a different level of oxygen exposure (Materials and Methods; S1 Fig). To ensure that any patterns we observed from PMA-seq were not procedural artifacts, we also sequenced PMA-seq controls that replaced PMA with water for some transplant preparations (see Materials and Methods; S2 Fig).

We found that total 16S rRNA abundance decreased with increasing exposure to oxygen, indicating that oxygen exposure decreases the number of viable cells (S3 Fig). This degradation was reflected in both untreated replicates—which captured DNA from living cells, dead cells, and free-floating DNA not associated with a cell—and replicates treated with PMA—which captured only DNA within living cells (S3 Fig).

**Fig 1:**
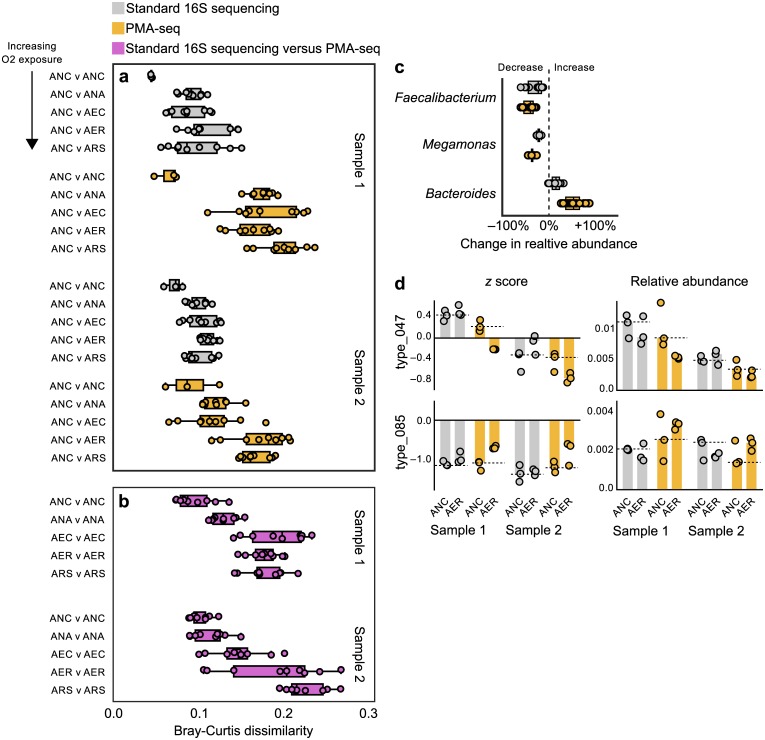
PMA-seq reveals changes in bacterial community composition with oxygen exposure. (a) Beta diversity (Bray-Curtis dissimilarity) between subsamples prepared with varying levels of oxygen exposure indicates that PMA-seq detects higher dissimilarity than standard 16S sequencing. (b) Beta diversity between standard 16S rRNA sequencing and PMA-seq results also reflects the degree of oxygen exposure. Data were generated from the same stool sample and same oxygen preparation, sequenced using either standard 16S rRNA sequencing or PMA-seq. (c–d) PMA-seq also detects changes in the abundance of individual OTUs to a greater extent than standard 16S sequencing. (c) OTUs from the genera *Faecalibacterium* and *Megamonas* largely decreased in relative abundance when exposed to oxygen, while those from *Bacteroides* increased. This signal was stronger in results from PMA-seq. Each point represents the mean change in relative abundance of a single OTU across three technical replicates. (d) Our analytical method identified individual OTUs that changed significantly in relative abundance between different oxygen preparations, many of which would have not been detected using standard 16S sequencing. Abbreviations of transplant preparation methods: ANC, anaerobic + cysteine; ANA, anaerobic; AEC, aerobic + cysteine; AER, aerobic; ARS, aerobic + sparging.

To understand which bacteria were affected, we analyzed 16S rRNA sequencing results. Standard 16S rRNA sequencing indicated a slight increase in beta diversity (Bray-Curtis dissimilarity) with increasing oxygen exposure, but these differences were much clearer in the PMA-seq data across all comparisons (Fig 1a, S4 Fig). Comparison with controls confirmed that the changes we observed were largely due to PMA's exclusion of unprotected DNA, not other steps in the PMA-seq process (S2 Fig). These results confirmed that PMA-seq more clearly reflects changes in bacterial composition due to differential oxygen exposure than does standard 16S sequencing.

Given that PMA-seq reflects only living bacteria, and standard 16S rRNA sequencing ought to more closely reflect the entire bacterial community, we hypothesized that comparing sequencing results from each of these methods might provide a proxy for how much a bacterial community has been degraded. We found that beta diversity values between 16S rRNA sequencing and PMA-seq results from the same subsample increased with greater oxygen exposure (Fig 1b). This result suggests that comparing standard 16S rRNA sequencing and PMA-seq results could provide a proxy for the degradation of living bacteria within fecal material.

**Fig 2:**
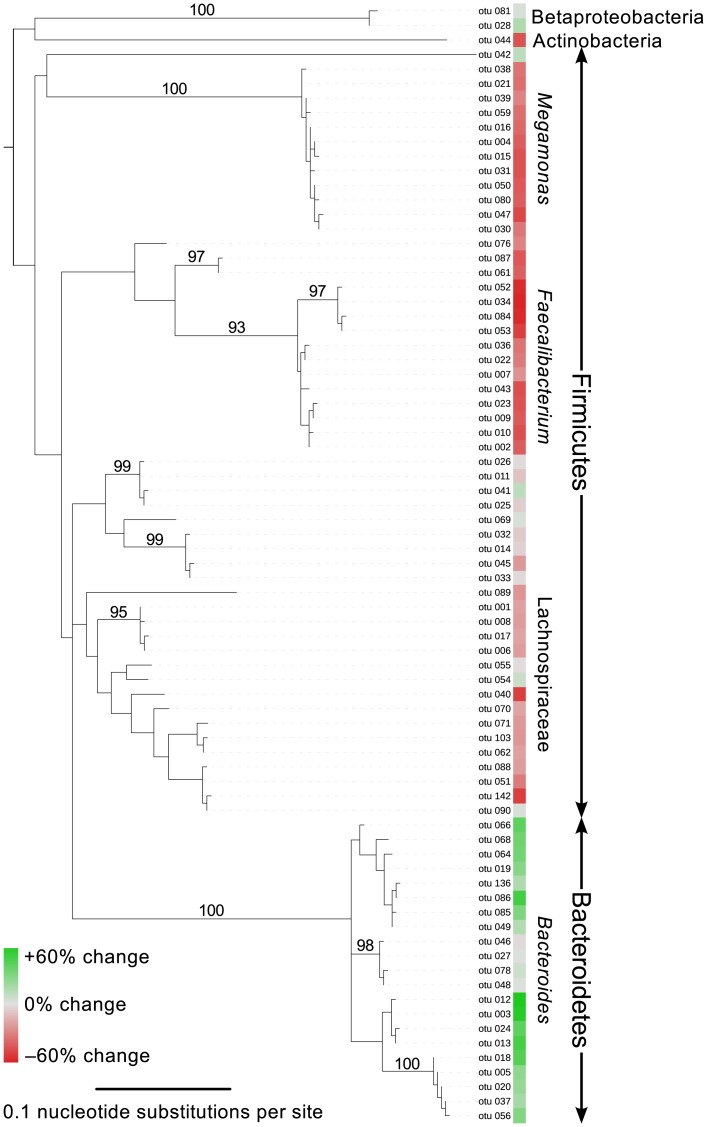
Responses to oxygen exposure cluster taxonomically. Phylogeny of high-confidence OTUs with their changes in abundance from ANC to AER transplant preparations. PMA-seq revealed clustered responses to oxygen exposure. Firmicutes, particularly those from *Megamonas* and *Faecalibacterium*, decreased in abundance with oxygen exposure, while those from *Bacteroides* increased. Branches with greater than 90% bootstrap support are annotated.

Our PMA-seq results also shed light on how specific bacterial taxa respond to short-term oxygen exposure, which could ultimately affect therapeutic efficacy. Oxygen appeared to have the greatest negative effect on the abundances of bacteria from the phylum Firmicutes (Fig 2). In particular, two of the four most abundant genera from one donor—*Megamonas* and *Faecalibacterium* (sp. *prausnitzii*)—uniformly decreased in abundance in both stool samples tested (Fig 1c, S4 Fig; comparison of anaerobic + cysteine and aerobic preparation, two-tailed Student's *t*-test, *t* = 6.293, *P* = 0.0033 and *t* = 7.494, *P* = 0.0017, respectively). Little is known about the role that *Megamonas* plays in the gut microbiota [26,27]. *Faecalibacterium prausnitzii* is believed to have anti-inflammatory properties in the gut and to help moderate or prevent illnesses like inflammatory bowel disease [28,29]. *F*. *prausnitzii* produces short-chain fatty acids, which help regulate host immune cells [30] and are the preferred energy source of colonic epithelial cells [31]. Thus, by decreasing the viability of *F*. *prausnitzii* cells during oxygen exposure, we may be compromising the therapeutic value of fecal transplant material.

We also identified oxygen-resistant bacteria—such as *Bacteroides*, *Parabacteroides*, Barnesiellaceae, and Rikenellaceae—that increased in relative abundance (Figs 1c and 2, S4 Fig). We hypothesized that these increases were compositional effects—which arise because we are measuring proportions rather than counting directly—since we expected little to no growth during our brief sample handling and freezer storage. Indeed, many of these apparent increases were flattened by normalizing the data to total community size (S3 Fig). OTUs from the genus *Bacteroides* were most often identified as oxygen resistant in our data, which aligns with previous evidence that some *Bacteroides* species can survive or even grow during short periods of oxygen exposure [32].

To identify individual OTUs that significantly changed in abundance between different transplant preparations, we created an analysis pipeline based on texmex [33], which models microbial community analyses using a Poisson log-normal distribution (see Materials and Methods, Fig 1d, S5 Fig). Using this pipeline and our table of high-confidence OTUs, we found that OTUs that decreased significantly in abundance during oxygen exposure most often belonged to the genera *Faecalibacterium*, *Megamonas*, and *Bifidobacterium*, mirroring the overall taxonomic shifts (S1 Table). OTUs that increased often belonged to *Bacteroides*, including *B*. *ovatus*, *B*. *uniformis*, and *B*. *caccae* (S1 Table).

Although responses to oxygen exposure by individual OTUs largely reflected the patterns of larger taxonomic groups, PMA-seq detected some heterogeneity in these responses (S6 Fig). For example, the most abundant OTUs within the genus *Oscillospira* did not all exhibit the same dynamics in response to oxygen exposure (S6 Fig).

### Freeze-thaw cycles and lag time have smaller effects on fecal bacterial composition

In addition to oxygen exposure during homogenization, we also used PMA-seq to evaluate the effects of freeze-thaw cycles and lag time—common concerns when working with gut microbiota samples. For freeze-thaw experiments, we prepared two stool samples according to our anaerobic + cysteine protocol (see Materials and Methods) and allowed them to freeze and thaw for the indicated number of cycles. For lag time experiments, we left subsamples of two stool samples in a biosafety cabinet for an allotted time before preparing them with our anaerobic + cysteine protocol. As we had done for oxygen exposure, we repeated each of these experiments with two separate stool samples from a single donor.

**Fig 3:**
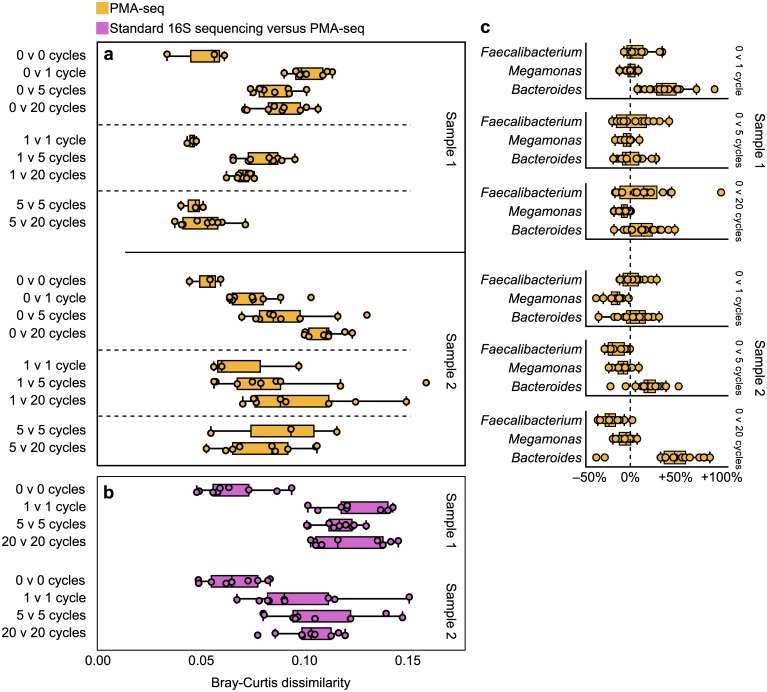
PMA-seq registers little alteration of the living bacterial community with more freeze-thaw cycles. (a) Beta diversity between subsamples from different freeze-thaw preparations reflected community perturbation, but the dissimilarity values were lower than for oxygen exposure. (b) Beta diversity between standard 16S rRNA sequencing and PMA-seq results reflected the number of freeze-thaw cycles. (c) OTUs of three dominant genera did not show uniform reactions to freeze-thaw cycles.

**Fig 4:**
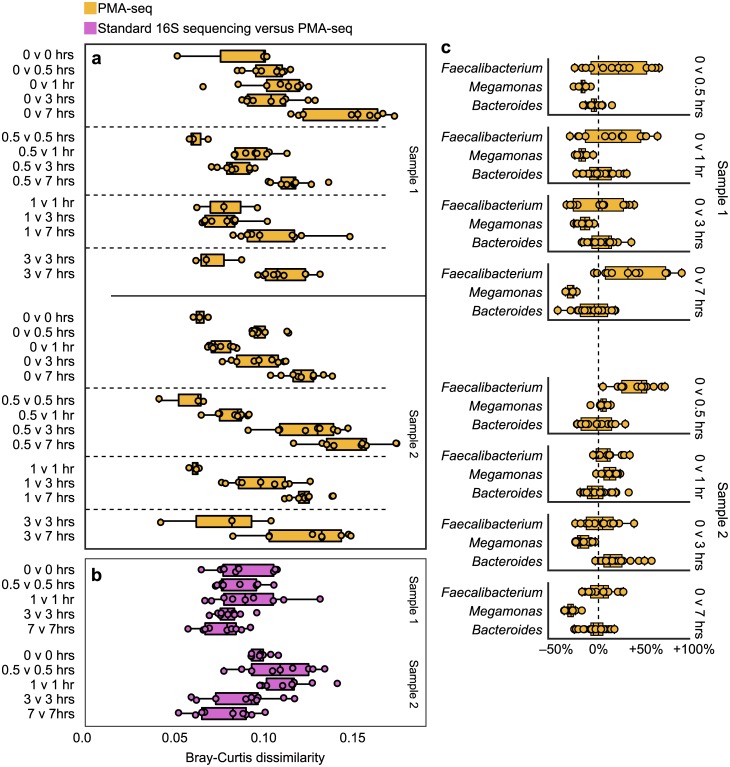
PMA-seq registers little alteration of the living bacterial community with longer lag time. (a) Beta diversity between subsamples from different lag time preparations also reflected community perturbation, but the dissimilarity values were lower than for oxygen exposure. (b) Beta diversity between standard 16S rRNA sequencing and PMA-seq results did not reflect longer lag times, suggesting that lag time did not greatly alter overall community composition. (c) OTUs of three dominant genera did not show uniform reactions to different lag times.

We found that bacterial community composition remained largely stable in response to freeze-thaw cycles (Fig 3, S7 Fig) and was not drastically altered by lag time (Fig 4, S8 Fig), even after as many as 20 freeze-thaw cycles or 7 hours of lag time. Beta diversity results from both experiments indicate that the communities as a whole did change with more freeze-thaw cycles (Fig 3a) and longer lag times (Fig 4a), but the changes with freeze-thaw cycles and lag time were smaller than those we observed in our oxygen exposure experiments (Fig 1). Beta diversity between 16S sequencing and PMA-seq results reflected the number of freeze-thaw cycles (Fig 3b) but not the duration of lag time (Fig 4b). These results further suggest that comparing these two sequencing methods might provide a proxy for overall community disturbance but may not capture all types of stress, particularly stresses affecting all bacteria equally, which would not be captured by measurements of relative abundance.

From our normalized data, it appears that freeze-thaw cycles had a small negative effect on the total abundance of living bacteria, with much of that effect occurring after one freeze-thaw cycle (S9 Fig). Our fecal microbiota preparations contained glycerol as a cryoprotectant, which ought to have dampened any effect freeze-thaw cycles would have had on bacterial composition and overall abundance. The lack of strong changes in community composition suggests that stress due to freeze-thaw cycles with this preparation method is less specific to certain taxa than is oxygen stress.

Longer lag times appeared to reduce total bacterial abundance (S10 Fig). We suspect that the increase in overall community size in stool sample 6 (S10 Fig) is an artifact of handling: since we left subsamples completely exposed in a biosafety cabinet, they dried considerably during the experiment, resulting in thicker, more rigid materials. We imagine that lag time had little effect on the composition of bacteria because this formation of a dried, stiff outer layer may have shielded the inner microbial community from the effects of oxygen, while degrading all microbes caught within the outer layer.

Reflecting our diversity analysis, few individual OTUs or taxa were reliably identified as significantly decreased or increased in abundance with different numbers of freeze-thaw cycles or length of lag time (S5 Fig, S1 Table). One OTU of *Bifidobacterium* did appear to decrease with freeze-thaw cycles, while some OTUs from *Faecalibacterium* and *Megamonas* were sensitive to lag time, potentially because of oxygen exposure (S1 Table). Our statistical methods revealed that the distributions of changes in abundance of individual OTUs between freeze-thaw and lag-time preparations resembled our null model, further indicating that the community was largely intact (S5 Fig). In contrast, the same distributions for different oxygen preparations diverged dramatically (S5 Fig), emphasizing a shift in community composition.

Our results suggest that moderate numbers of freeze-thaw cycles and moderate lag times—particularly if stool samples are covered—do not alter fecal microbial communities as much as oxygen exposure during homogenization and may in fact not greatly affect therapeutic efficacy.

## Discussion

Although previous studies have evaluated the effects of various collection and storage methods on 16S rRNA extraction [34–37], to our knowledge, ours is the first analysis of how these variables alter the representation of living bacteria. Our results confirm a common assumption that oxygen exposure compromises the composition of viable fecal microbes [3,38,39]; they also support the notion that moderate numbers of freeze-thaw cycles and lag-time may only minimally damage the microbial community. Oxygen exposure disproportionately affected bacteria from the phylum Firmicutes—particularly the genera *Megamonas* and *Faecalibacterium*—while bacteria from the genus *Bacteroides* appeared to be more oxygen-tolerant (Fig 2). Members of all three genera are generally thought to be strictly anaerobic [28,32,40], suggesting that information on growth conditions alone may be insufficient to predict how bacterial taxa in complex communities will respond to stress.

At this time, it is unknown whether these alterations of the microbial community might alter therapeutic potential. On the one hand, current practices of preparing fecal transplants to treat *C*. *difficile* infections involve processing in aerobic conditions [3,39] and freezing fecal material [39,41,42]. Treatment nevertheless typically succeeds, making it unlikely that these practices alter therapeutic efficacy for this infection. On the other hand, some of the bacteria most negatively affected by oxygen exposure included the anti-inflammatory bacterium *F*. *prausnitzii* [28]. If *F*. *prausnitzii* has therapeutic effects—as has been posited for inflammatory bowel disease [28,29]—aerobic processing of fecal transplants may decrease therapeutic efficacy for treating inflammatory diseases.

Degradation of the microbial community with increasing oxygen exposure indicates that therapeutic efficacy for fecal microbiota transplants—particularly in diseases other than *C*. *difficile* infection—may be best preserved by maintaining anaerobic conditions during sample processing (for example, by using anaerobic chambers and oxygen-free buffers) and storage (for example, by using oxygen-impermeable containers and monitoring for oxygen exposure). The relatively smaller effects we observed from freeze-thaw cycles and lag time suggest that flexibility in these aspects of fecal processing and delivery might not greatly affect downstream efficacy.

Our experiments on preparation methods for fecal microbiota transplants suggest that PMA-seq could provide a valuable tool to assess how to identify, prepare, and administer such transplants for recurrent *C*. *difficile* infections and other indications. Other methods to quantify bacterial communities—such as 16S-rRNA sequencing, qPCR, or fluorescence microscopy—either fail to identify individual bacterial taxa or fail to target only living cells, which are presumably the therapeutic agent of fecal microbiota transplants. By addressing these shortcomings, PMA-seq could provide quality control and reduce sources of variability in fecal microbiota transplants.

## Materials and Methods

### Sample preparation

To prepare fecal microbiota transplants for PMA-seq analysis, we used modified protocols based on standard practices at OpenBiome (http://www.openbiome.org/), the largest stool bank in the United States. All human stool collections and subject consent procedures were reviewed and approved by the Institutional Review Board of the Massachusetts Institute of Technology, approval number 1510271631. All participants provided written consent.

To examine how oxygen exposure alters the fecal microbial community, we used PMA-seq to evaluate stool samples processed in different oxygen conditions. We prepared two stool samples from a single donor with five different fecal microbiota–preparation protocols with varying levels of oxygen exposure (S1 Fig). We transferred each stool sample into an anaerobic chamber (Coy) within 30 min of passage. We split the stool into four 30 g subsamples. For each of these four subsamples, we prepared the fecal microbiota transplant by varying two factors: the buffer used to homogenize the stool and the environment in which the stool was homogenized. We used two similar buffers: one of 50% glycerol, 50% saline solution (0.9% NaCl), and 0.1% L-cysteine buffer and another of 50% glycerol plus 50% saline solution. L-cysteine is a reducing agent, which reacts with oxygen to remove it from solution. For the homogenization environment, we homogenized the stool either within the anaerobic chamber or in ambient aerobic conditions. For subsamples prepared in anaerobic conditions, we prereduced each homogenization buffer by leaving it in the anaerobic chamber for at least 48 hr. This procedure led to four different preparations: anaerobic + cysteine, anaerobic, aerobic + cysteine, and aerobic. For the final preparation (aerobic + sparging), we then placed half the aerobic fecal homogenate into a sealed glass media bottle and sparged the homogenate with air for 30 min.

We homogenized all 30 g subsamples in separate Whirl-Pak filter bags (Nasco) with 150 ml buffer. We transferred each filter bag into an easyMix automated homogenizer (AES Chemunex) and homogenized the contents for 60 s. We then transferred aliquots of 498.75 μl of stool homogenate into screw-cap tubes, which we sealed in secondary screw-top glass containers and froze at –80°C to await further processing.

To test the effects of freeze-thaw cycles on fecal bacteria, we subjected fecal microbiota transplants to 0, 1, 5, and 20 freeze-thaw cycles. For this experiment, we prepared two stool samples from the same donor, following our anaerobic + cysteine protocol. We prepared six tubes of fecal homogenate from each of these stool samples as our zero freeze-thaw-cycles subsample preparation, and the rest we transferred to a freezer at –80°C for at least 3 h. We then allowed these subsamples to thaw at room temperature for 30 min before returning them to the freezer for 3 h for the indicated number of cycles.

We also tested the effects of lag time on the fecal microbiota. For these experiments, we transferred each of two stool samples to an anaerobic chamber and split each sample into five subsamples of 50 g each. One subsample was processed immediately as a zero time point. We removed the remaining subsamples from the anaerobic chamber and transferred them to a biosafety cabinet. We then prepared each subsample after the indicated exposure time had lapsed (0.5, 1, 3, and 7 h; 1 h is a standard limit at OpenBiome). At that time, we then prepared each subsample according to our anaerobic + cysteine protocol.

### PMA to exclude unprotected DNA

To identify the living bacteria in each fecal microbiota preparation from all three experiments, we used PMA to exclude DNA from bacteria with compromised membrane structure, using the manufacturer's suggested protocol. We further divided subsamples from all experiments into six technical replicates, three of which we analyzed using PMA-seq and three of which we analyzed using standard 16S sequencing. For the PMA-seq replicates, we added 1.25 μl of 20 mM PMA dye (Biotium) to each aliquot of stool homogenate, to a final volume of 500 μl and final concentration of 50 μM PMA. We covered aliquots in aluminum foil and incubated them at room temperature for 5 min, vortexing every minute. We then removed the aluminum foil and photolysed the aliquots on ice under an LED light (Taotronics TT-AL09) for 30 min, rotating them every 10 min. After photolysis, we extracted DNA using a PowerSoil DNA extraction kit (MoBio). In parallel, we also extracted DNA from unaltered aliquots of fecal homogenate for standard 16S rRNA sequencing. To ensure that any signal we observed in the PMA-seq results did not come from incubation and photolysis, we also ran controls that replaced 1.25 μl of 20 mM PMA dye with water and underwent the same procedure as for PMA-seq.

### Illumina library preparation and analysis

From these PMA-treated and untreated DNA samples, we then prepared 16S rRNA libraries using a two-step PCR protocol [22] to identify bacterial community composition. First, we quantified extracted DNA concentrations using a standard SYBR Green qPCR protocol with Phusion polymerase (New England Biolabs; used for all PCR reactions) and primers PE16S_V4_U515_F and PE16S_V4_E786_R (S2 Table). We diluted all DNA samples to the concentration of the most dilute DNA sample and used 2 μl of each DNA sample for a PCR reaction with primers PE16S_V4_U515_F and PE16S_V4_E786_R (S2 Table) and a program of 98°C for 30 s [98°C for 30 s, 52°C for 30 s, 72°C for 30 s] for 20 cycles, 4°C hold. For each DNA sample, we ran four 25 μl PCR reactions, which we then pooled, cleaned using SPRI AmpureXP beads, and eluted in 40 μl of elution buffer. For the second PCR reaction, we used 4 μl of the previous PCR product with primers PE-PCR-III-F and PE-PCR-IV-barcode in four 25 μl reactions with a PCR cycle of 98°C for 30 s [98°C for 30 s, 83°C for 30 s, 72°C for 30 s] for 7 cycles, 4°C hold. We pooled each set of four PCR reactions and cleaned the reactions using SPRI beads. We quantified library concentrations using another SYBR Green qPCR with primers BMC Final F and R (S2 Table). We multiplexed DNA libraries so that they had equal DNA input. Libraries were sequenced on a single Illumina MiSeq lane set for paired-end, 250-base-pair reads.

We cleaned, merged, and filtered raw paired-end sequence reads using default parameters in UPARSE [43] and clustered the data into OTUs using a 97% identity threshold in QIIME [44]. We used default QIIME settings to remove chimera sequences [45], pick *de novo* OTUs [46], and assign taxonomy [47,48]. We assigned taxonomy to each OTU on the basis of the August 2013 release of the Greengenes rDNA database [47]. We excluded OTUs that did not appear in at least two samples. We used qPCR results from the initial qPCR of each DNA sample to normalize our relative-abundance results.

### Identifying OTUs that changed significantly in abundance

To identify OTUs that differed significantly in abundance between transplant preparations, we built an analysis pipeline that involved two primary steps: first, fitting OTU abundance data from each DNA sample to the Poisson log-normal distribution, which allows for cross comparisons, and second, fitting these comparisons to a generalized normal distribution, which provides a null model against which we could compare, and thereby identify, OTUs with significant changes in abundance.

For the first analysis step, we started by creating an OTU table with high-confidence OTUs. To create this table, we first clustered sequencing data using DADA2 and default parameters and procedures [49]. We then filtered for OTUs that were present in all DNA samples, resulting in a table of 77 high-confidence OTUs, where the rarest OTU represented 0.15% of all reads. We fit OTU abundances from individual DNA samples to a Poisson log-normal distribution using pytexmex, a Python implementation of texmex [33]. Previous studies have found that the Poisson log-normal distribution is an appropriate statistical model for microbial communities in many different habitats, including the human microbiome [33].

Using this model, we then calculated two metrics for each OTU, *z* and *F*, which could then be compared across DNA samples to evaluate changes in an OTU's abundance in one sample compared with another. The *z* metric reflects each OTU's normalized abundance within a Poisson log-normal framework. The *F* metric reflects how each OTU's abundance compares with the abundances of all other OTUs in that DNA sample (i.e., the OTU's position in the Poisson log-normal distribution for that DNA sample). For a given OTU, comparisons of these metrics (Δ*z* and Δ*F*) between two DNA samples thus reflected the change in an OTU's normalized abundance and the change in an OTU's position within the distribution of abundances from each sample [33].

In the second analysis step, we sought to build a statistical framework to identify OTUs with putatively significant Δ*z* and Δ*F* metrics between different transplant preparations. To build our null model, we used variation between technical replicates of the same transplant preparation and the same stool sample. Thus, for a given pair of transplant preparations (from the same stool sample), we first calculated all possible Δ*z* and Δ*F* values—for every OTU—among technical replicates of each preparation. We then fit these values to a generalized normal distribution (S5 Fig) to make our "null model" of values expected from technical variation but not biological variation.

To identify OTUs whose Δ*z* and Δ*F* values were significantly greater or less than values expected from technical variation, we then calculated Δ*z* and Δ*F* values for each OTU across transplant preparations. We considered those OTUs whose Δ*z* and Δ*F* values fell outside of 95% of the null model distribution—values that had only a 5% chance of occurring in comparisons of technical replicates—as putatively significant (S5 Fig). We further trimmed these OTUs to include only OTUs that had significantly greater (or smaller) Δ*z* and Δ*F* values in all pairwise replicate comparisons. We identified these final OTUs as changed significantly in abundance. Values of Δ*z* were generally not affected by the overall abundance of each OTU (S11 Fig), but Δ*F* values were (S12 Fig), meaning that Δ*F* values may be more prone to bias.

Data can be accessed on the US National Center for Biotechnology Information SRA database under BioSample SAMN04962333.

## Supporting Information

S1 Fig Experimental design for oxygen exposure experiments. (EPS)

S2 Fig PMA-seq H_2_O controls verify that PMA, not other PMA-seq processing steps, alters 16S sequencing results. (a) Beta diversity between H_2_O controls, standard 16S sequencing, and PMA-seq results. (b) Stacked bar plots and (c) individual bar plots of relative abundance of bacterial genera in anaerobic + cysteine and aerobic preparations from data generated using standard 16S sequencing, PMA-seq, and H_2_O controls.
(EPS)

S3 Fig Normalized abundances of bacterial taxa with increasing oxygen exposure. Stacked bar plots of relative abundances are normalized to total community size, which we estimated with qPCR.
(EPS)

S4 Fig Raw abundance data for oxygen exposure experiments. Data from two stool samples prepared with varying degrees of oxygen exposure and analyzed using standard 16S sequencing or PMA-seq. (a) Stacked bar plots and (b) individual bar plots of relative abundances of different bacterial genera observed with both sequencing methods from stool sample 1; (c) and (d) show results from stool sample 2.
(EPS)

S5 Fig Using the Poisson log-normal distribution to identify OTUs that changed significantly in relative abundance. Histograms of (a) Δ*z* and (b) Δ*F* scores of OTUs calculated between technical replicates (cyan) and across oxygen, freeze-thaw, and lag time preparations (red). Fit lines are generalized normal distributions. Dotted blue lines indicate the 0.05 cutoff for the distribution of Δ*z* and Δ*F* scores between technical replicates. In the case of oxygen exposure, the distribution of Δ*z* and Δ*F* scores between transplant preparations was much wider than the distribution of Δ*z* and Δ*F* scores between technical replicates, indicating pervasive shifts in abundance. In contrast, the same distributions for freeze-thaw and lag time preparations look very similar.
(EPS)

S6 Fig Individual OTUs within bacterial genera showed variable responses to oxygen exposure. Log-transformed relative abundances of the five most abundant OTUs from common bacterial taxa. Error bars represent standard error. Although OTUs within the genera *Faecalibacterium* and *Megamonas* responded similarly to oxygen, we observed different responses from OTUs within *Ruminococcus*, and *Oscillospira*.
(EPS)

S7 Fig PMA-seq registers little alteration of the living bacterial community with more freeze-thaw cycles. (a) Stacked bar plots and (b) individual bar plots of the relative abundances of various bacterial genera with more freeze thaw-cycles, as identified by PMA-seq.
(EPS)

S8 Fig PMA-seq registers little alteration of the living bacterial community with longer lag times. (a) Stacked bar plots and (b) individual bar plots of the relative abundances of various bacterial genera with longer lag times, as identified by PMA-seq.
(EPS)

S9 Fig Normalized abundances of bacterial taxa with more freeze-thaw cycles. Stacked bar plots of relative abundances are normalized to total community size, which we estimated with qPCR.
(EPS)

S10 Fig Normalized abundances of bacterial taxa with longer lag times. Stacked bar plots of relative abundances are normalized to total community size, which we estimated with qPCR.
(EPS)

S11 Fig Δ*z* scores are largely unaffected by sequencing depth. Mean Δ*z* scores for each OTU plotted against the mean abundance for that OTU. Data shown are from six transplant preparation comparisons from two stool samples.
(EPS)

S12 Fig Δ*F* scores appear to be affected by sequencing depth. Mean Δ*F* scores for each OTU plotted against the mean abundance for that OTU. Data shown are from six transplant preparation comparisons from two stool samples.
(EPS)

S1 Table Taxonomic information of OTUs identified as differentially abundant between transplant preparations according to our Poisson log-normal analysis pipeline. (XLSX)

S2 Table Primers used in this study. (XLSX)

S1 Data OTU table with OTUs clustered at 97% similarity. (GZ)

S2 Data OTU table of 77 high-confidence OTUs—ones present in all samples—clustered at 100% similarity. (GZ)

S3 Data Sample metadata. (GZ)
